# Identification of Novel Alternative Splicing Events Associated With Tumorigenesis, Protein Modification, and Immune Microenvironment in Early-Onset Gastric Cancer

**DOI:** 10.3389/fonc.2021.640272

**Published:** 2021-06-08

**Authors:** Jian Zhang, Ajay Goel, Lin Zhu

**Affiliations:** ^1^ Department of Pharmaceutical Sciences, Irma Lerma Rangel College of Pharmacy, Texas A&M University, College Station, TX, United States; ^2^ Beckman Research Institute, City of Hope Comprehensive Cancer Center, Biomedical Research Center, Monrovia, CA, United States

**Keywords:** alternative splicing, immune microenvironment, early-onset gastric cancer, protein phosphorylation, protein glycosylation, alternative polyadenylation

## Abstract

**Background:**

Alternative splicing (AS), e.g. the tandem alternative polyadenylation (TAPA), has emerged as major post-transcriptional modification events in human disease. However, the roles of the AS and TAPA in early-onset gastric cancer (EOGC) have not been revealed.

**Methods:**

The global AS profiles of 80 EOGC patients were analyzed. The EOGC-specific AS events (ESASs) were identified in both the EOGC and adjacent non-tumor tissues. The functional enrichment analysis, Splicing network, Alternative Polyadenylation (APA) core factor network, and cell abundancy analysis were performed. Furthermore, the landscapes of the AS events in the varied subtypes of the EOGC patients were evaluated.

**Results:**

Overall, 66,075 AS events and 267 ESASs were identified in the EOGC. Furthermore, 4809 genes and 6152 gene isoforms were found to be aberrantly expressed in the EOGC. The Gene Ontology (GO) and Kyoto Encyclopedia of Gene and Genome (KEGG) pathway analyses showed that the significant pathway alterations might exist in these AS events, genes, and gene isoforms. Moreover, the Protein-protein interaction (PPI) network analysis revealed that the UBC, NEK2, EPHB2, and DCTN1 genes were the hub genes in the AS events in the EOGC. The immune cell infiltration analysis indicated a correlation between the AS events and the cancer immune microenvironment. The distribution of the AS events in varied EOGC subtypes, protein phosphorylation and glycosylation was uneven.

**Conclusion:**

The study highlighted the vital roles of the AS in the EOGC, including modulating the specific protein modification and reshaping the cancer immune microenvironment, and yielded new insights into the diagnosis of the EOGC as well as cancer treatment.

## Introduction

Gastric cancer (GC), a morbid and frequently lethal malignancy, is one of the most common cancers and the leading cause of cancer death worldwide, especially in East Asia (1). For most patients, the GC is usually associated with the unfavorable prognoses and can be only diagnosed at the relatively late stages, resulting in the limited treatment options. The major types of cancer, including the brain, cervix, esophageal squamous cell carcinoma, Kaposi sarcoma, larynx, lung, and non-Hodgkin lymphoma, remain a relatively low incidence rate among young adults. However, the cases of gastric non-cardia cancer in young adults kept increasing from 1995 to 2014 in the US (2). The GC occurring in the patients under the age of 45 is defined as EOGC (3). Though the genetic and environmental factors have been identified to be associated with the GC, the occurrence of EOGC remains largely unexplained.

Studies have profiled the alternative splicing (AS) events in The Cancer Genome Atlas (TCGA) gastric carcinomas (4), Epstein-Barr virus (EBV) associated gastric carcinomas (5), and gastric cell lines (6, 7), which ascertained the important roles of the AS events in the GC. Other accumulating evidence also show that the somatic CDH1 or TGFBR1 gene mutation and proteogenomic alteration are remarkable and may unleash the GC in younger adults (8, 9), suggesting the importance of the post-transcriptional regulation in the EOGC. The AS, one of the key post-transcriptional events, can generate various mRNA transcripts (isoforms) and affect their stability as well. As a result, the downstream protein variants translated from these mRNA isoforms vary in their sequences (10). On the one hand, the protein synthesis is directed by the human genome, but on the other hand, the protein diversity is ensured to satisfy various biological processes (11). Li et al. found the novel AS events and peptides during the mouse stomach formation (12). Furthermore, a comparative genomic study has been performed and identified a distinct molecular expression profile in the EOGC rather than the late onset gastric cancer (LOGC) (13). All these studies suggest that EOGC may have a varied AS event landscape compared to other types of GC.

The TAPA event introduces two or more poly(A) sites to the 3′ UTR of a gene which is distributed across a wide variety of cancers and modulates the sensitivity of certain anticancer drugs (14, 15). The shift of the TAPA events shows a cancer-specific and cell organelle-specific mode (16). It was reported that the APA-guided shortening of the NET1 gene 3’ UTR enhanced the mRNA transcriptional activity and promoted the metastasis of gastric cancer cells (17). Some studies defined the TAPA as a subtype of AS, while others consider it a pre-mRNA processing (18). In this study, the TAPA was analyzed as the AS.

So far, only a few genome-wide association studies on the EOGC have been performed and none of them have taken into account the effects of the AS events on the EOGC. To our knowledge, the role of the AS in the EOGC remains vogue. Herein, the major aims of this study are to analyze the AS events including the TAPA events and to evaluate their roles in the EOGC. The study contains the large-scale RNA sequencing data generated from the EOGC samples. The human transcriptome is surveyed to identify the genome-wide AS events in the EOGC and the ESAS are identified thereafter. Furthermore, the biological functions of these events are explored. The AS events in different EOGC subtypes are elucidated and their association with the molecular features and tumor immune microenvironment are also profiled.

## Methods

### Data Source

The RNA-Seq data of an independent cohort (tumor tissues and adjacent normal tissues from 80 EOGC patients) was retrieved from the European Nucleotide Archive (study accession: PRJNA508414). One adjacent normal tissue sample (SRR8281377) was excluded from this dataset due to the file error when the Whippet software read and processed the data. These patients were histologically diagnosed as GC and with the age at the time of surgery ≤ 45 years.

### RNA-Seq Analysis of the AS Events

Paired-end reads per sample data were generated using the HiSeq 2500 platform sequencer (Illumina). The fastq file was processed by Whippet (version 0.11) on our Linux system (19) and aligned to the Homo sapiens GRCh37.75 genome assembly (ftp://ftp.ensembl.org/pub/release-75//fasta/homo_sapiens/dna/Homo_sapiens.GRCh37.75.dna.primary_assembly.fa.gz). Besides default settings, Whippet was run with the “biascorrect” option to implement the GC-content and 5’ sequence bias correction. The Percent Spliced-in Index (PSI or ψ) value for the AS events as well as read count of genes and gene isoforms were generated by Whippet. The output file (diff. format) for the comparative analysis of PSI was generated by Whippet-delta.jl using default parameters. The comparative analysis of genes and gene isoforms was computed by the limma (20) and/or edgeR (21) package.

The PSI value (ranging from 0.0 to 1.0) was defined as the proportional abundance of certain AS events and was calculated for the eight types of the AS events. The AS events were determined using the maximum likelihood estimation by the expectation-maximization (EM) algorithm. To generate more accurate AS event profiling, the stringent filters (percentage of samples with PSI values > 95%, minimum PSI value > 0.05) were implemented. For the gene and gene isoform profiles, genes or gene isoforms with the minimum Counts Per Million (CPM) > 0.5 and percentage of samples with the read count values > 95% were included.

### Identification and Pathway Analysis of the ESAS, Genes, and Gene Isoforms

To filter the ESAS in the gastric cancer, the PSI values of the AS events were compared between the tumor and the matched adjacent normal tissues. The AS events with the missing data exceeding 90% of all subjects were excluded. Statistical analysis was performed in the differentially expressed AS events with > 0.9 probability and absolute log2 fold change ≥ 1. The genes or gene isoforms with the absolute log2 fold change ≥1 and false discovery rate (FDR) < 0.05 were defined as the EOGC-specific genes or gene isoforms. Interactive sets among the eight types of the AS were visualized by UpSetR (version 1.4.4) (22).

The associations between the parent genes of the ESAS and biological annotation terms (Gene Ontology [GO] and Kyoto Encyclopedia of Gene and Genome [KEGG] pathway) were detected using the clusterProfiler package (3.16.0) (23). The NetworkAnalyst updated on 05/2020 was applied to analyze the parent genes of each ESAS for constructing the visualized PPI network (24). The STRING Interactome was selected in the PPI database (confidence score cut-off value 900).

### Immune Cell Infiltration and ESAS Event Analysis

The differential gene expression between the tumor and normal tissues and the percentage of the cell abundance in the TCGA STAD cohort were calculated by TIMER 2.0 (25). The gene expression data from our study was input into ImmuCellAI (26) to impute the abundance of 24 types of immune cells in the EOGC. Spearman’s rank correlation analysis was used to calculate the correlations between the PSI values of the ESAS and immune cell type. The threshold of Spearman’s rank correlation coefficients was set to > 0.4 or < -0.4, and the BH adjusted p-value < 0.05.

### Construction of Correlation Network Among the Splicing Factors, APA Core Factors, and ESAS Events

From the database (27) and publication (28), we selected 71 experimentally validated human splicing factors and 22 APA core factors to build the splicing factor correlation network and APA core factor correlation network. Spearman’s rank correlation analysis was used to impute the correlations between the expression of splicing factors or APA core factors and PSI values of ESASs or TAPAs. The threshold of Spearman’s rank correlation coefficients was set to > 0.4 or < -0.4, and the BH adjusted p-value < 0.05. The correlation network was constructed and visualized by Cytoscape (version 3.7) (29). All statistical analyses were performed by the R language (30) and p-value < 0.05 was considered statistically significant unless specified.

### Identification of the EOGC Subtype Relevant AS Events

The data of the EOGC EBV status, microsatellite instability (MSI) status, protein phosphorylation subtype, and protein glycosylation subtype were accessed from Mun et al. (9). The AS events which have > 0.9 probability of being differentially expressed among the different EBV status, MSI status, protein phosphorylation subtypes, and protein glycosylation subtypes were defined as the subtype relevant AS events in the EOGC. The groups with the sample numbers of less than four were not included in the analysis. The PSI difference among the groups was required to be larger or equal 0.1 for at least two groups to ensuring the biological significance. The analysis was performed for each subtype separately.

## Results

### Overview of the AS Events in the EOGC Cohort

**Figure 1:**
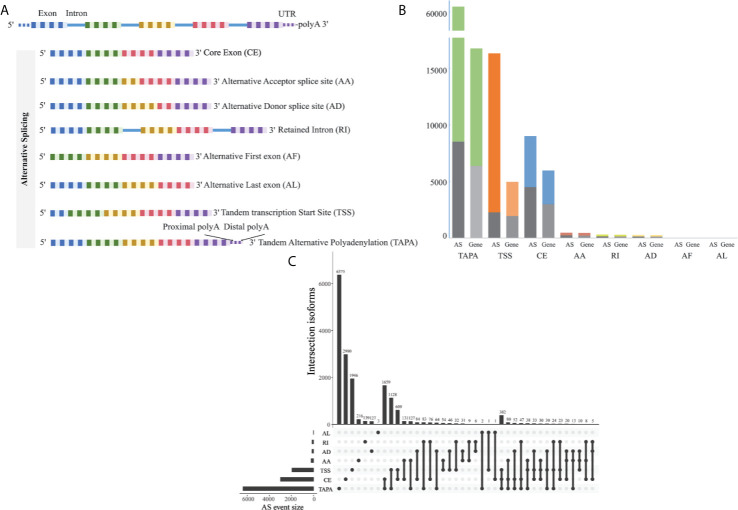
Profiling of the AS events in the EOGC. **(A)** Scheme of six canonical and two novel patterns of the AS events: CE, AA, AD, RI, AF, and AL, TSS and TAPA; **(B)** Number of the AS events and their parent genes in the EOGC patients. The bar color represents the filtered AS events and their parent genes. The black bars represent the AS events and their parent genes filtered using the stringent filters; **(C)** Interactive sets among seven patterns of the AS events (n = 15,803) shown in an UpSet plot.

The corresponding RNA-Seq data of 80 EOGC patients were used to establish the integrated AS event profiling. We identified 66,075 AS events from 11,282 genes, which accounted for 53.0% of non-redundant human protein-coding genes (31). Beside two special patterns of the AS events including the tandem transcription start site (TSS) and tandem alternative polyadenylation site (TAPA), these AS events were classified into six canonical splicing patterns: core exon (CE), alternative acceptor splice site (AA), alternative donor splice site (AD), retained intron (RI), alternative first exon (AF), and alternative last exon (AL), as illustrated in **Figure 1A**. Among these splicing patterns, the TAPA occurred most frequently (71.0%) (**Figure 1B**).

Some gene isoforms showed very low redundancy, thus, we screened the AS events with a series of filters (percentage of samples with PSI values ≥ 75%, PSI value ≥ 0.05). Consequently, a total of 15,803 AS events from 8,359 genes were obtained. After filtering, the AF events were excluded. The analysis indicated that the TAPA was still the top AS pattern (54.0%) (**Figure 1C**). After removal of the duplicated genes in one AS pattern, the Upset plots were created to quantitatively analyze the interactive sets of the remaining seven patterns of the AS events. As shown in **Figure 1C**, most of the genes had more than one pattern of the AS events, among which some genes had up to three different splicing patterns.

### Identification of the ESAS Events

**Figure 2:**
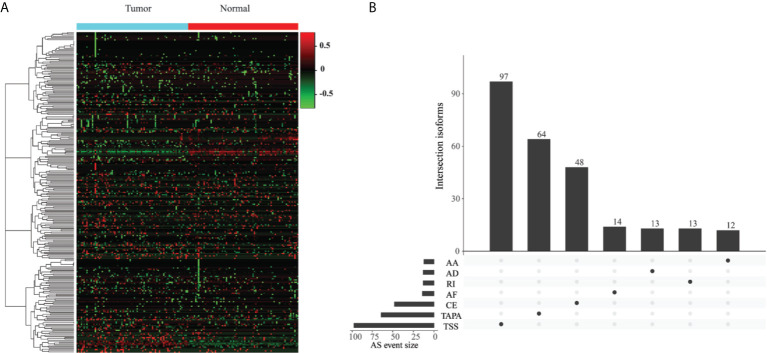
Identification of the EOGC-specific AS events. **(A)** Heatmap of the ESAS between 80 paired tumor tissues and 79 adjacent normal tissues (absolute fold change ≥ 0.1, probability > 0.9); **(B)** The landscape of 267 ESAS shown in an UpSet plot.

To identify the EOGC-specific AS events, we compared the PSI values between 80 paired tumor tissues and 79 adjacent normal tissues. A total of 267 EOGC-specific AS events (ESASs) from 228 genes were identified (**Figure 2A** and **Table S1**). Although a large number of the AS events were detected in the EOGC cohort, a relatively small proportion of the AS events were identified as the ESAS. The TSS and TAPA events accounted for 36.3% and 24.0% of the ESAS, respectively (**Figure 2B**). The uneven distribution of the AS patterns in the tumor and adjacent normal tissues indicated that they might play important roles in the early-onset gastric tumorigenesis.

For the single gene with multiple ESAS events, the regulatory directions (up- or down-regulation) of varied ESAS events between the tumor and normal tissues can be either the same or opposite. Eight genes (COL5A1, CBWD1, SLC47A2, SSPO, AL020989.1, RPL5P1, AACSP1, and AC022905.1) exhibited the same regulatory direction of AS events; while the other 22 genes showed the opposite regulatory direction (**Table S2**). Interestingly, the proportions of certain AS patterns between the ESAS and entire AS events were inconsistent. The TAPA, a top pattern among all AS events (54.0%), only contributed to 24.0% of the ESAS events.

### Profile of the EOGC-Specific Genes and Gene Isoforms

Based on the above results, we further studied how the dysregulated AS events affected the expressions of the genes and gene isoforms. We identified 4809 genes from a total of 16383 genes, and also identified 6152 gene isoforms from a total of 16283 gene isoforms (CPM > 0.5 and the percentage of samples with read count values > 95%).

**Figure 3:**
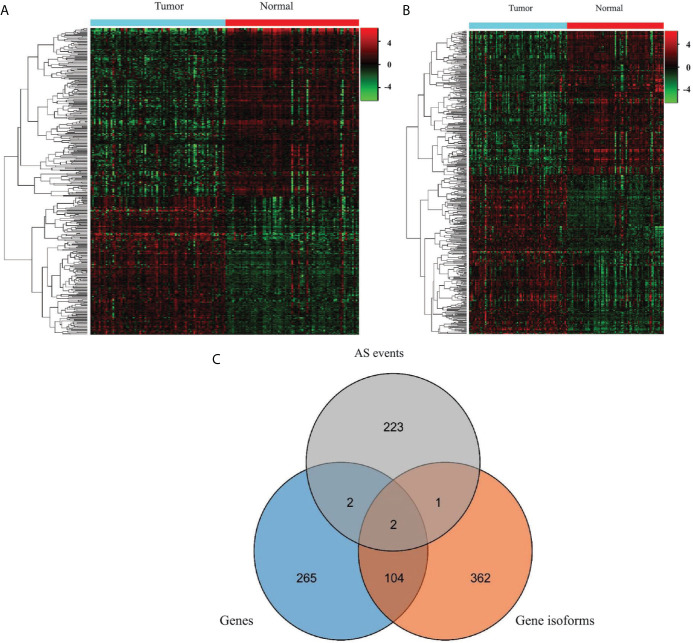
Identification of the EOGC-specific genes and gene isoforms. **(A)** Heatmap of the EOGC-specific genes between 80 paired tumor tissues and 79 adjacent normal tissues (absolute fold change ≥ 1, FDR < 0.05); **(B)** Heatmap of the EOGC-specific gene isoforms between 80 paired tumor tissues and 79 adjacent normal tissues (absolute fold change ≥ 1, FDR < 0.05); **(C)** Illustration of the intersection set of the ESAS, EOGC-specific genes, and gene isoforms by the Venn diagram.

To generate EOGC-specific genes and gene isoforms, we compared the read count value of the genes or gene isoforms from the tumor and normal or adjacent non-tumor tissues by the limma-voom (32). After refining, 373 genes and 469 gene isoforms were identified as the EOGC-specific genes and gene isoforms, respectively (absolute log2 fold change ≥1 and FDR < 0.05, **Figures 3A, B** and **Table S3**). In addition, we profiled the enriched TF binding motifs in the promoters of the EOGC-specific genes and gene isoforms (**Table S4**). A total of four genes and three gene isoforms with ESASs were differentially expressed in the EOGC (**Figure 3C**).

### Pathway and Protein-Protein Interaction (PPI) Network of the ESAS Events and EOGC-Specific Genes and Gene Isoforms

**Figure 4:**
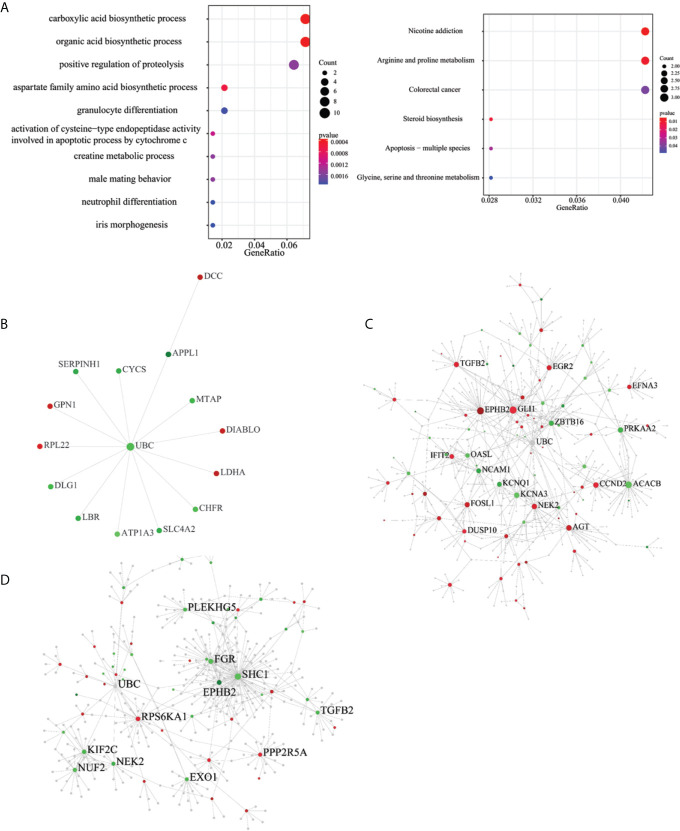
Pathway and protein-protein interaction (PPI) network of the ESAS events, EOGC-specific genes, and gene isoforms. **(A)** Top 10 GO and KEGG pathways with a P-value < 0.05; **(B)** Protein-protein interaction network of the ESAS events (Zero-order network); **(C, D)** Protein-protein interaction network of the EOGC-specific genes and gene isoforms, respectively (first-order network). Red: up-regulation; Green: down-regulation.

To understand the biological roles of the ESAS and EOGC-specific genes and gene isoforms, the GO and KEGG pathway analyses were performed. The results showed that the ESAS parent genes were linked to the GO terms of carboxylic acid biosynthetic process (GO:0046394), organic acid biosynthetic process (GO:0016053), purine-containing compound metabolic process (GO:0072521), collagen-containing extracellular matrix (GO:0062023), etc. These ESAS parent genes were also found to be enriched in the KEGG signaling pathways, including Nicotine addiction (hsa05033) and Arginine and proline metabolism (hsa00330) (**Figure 4A** and **Table S5**). The GO and KEGG pathway results of the EOGC-specific genes and gene isoforms were also included in the supplementary table (**Table S5**).

The pathway analysis results showed that the parent genes of ESAS might play a vital role in regulating the cancer-related biological processes. The parent genes of the ESAS were further analyzed by the PPI network. Using the Zero-order network, we built the PPI network of the ESAS parent genes, which contained 15 nodes, 14 edges and 15 seeds (**Figure 4B** and **Table S6**). We found that the UBC was the hub gene in the network. Meanwhile, based on the first-order network, we also built 29 and 27 PPI networks using the EOGC-specific genes and gene isoforms, respectively (**Figures 4C, D** and **Table S6**). The UBC, NEK2, EPHB2, and DCTN1 genes were identified as the hub genes in two or more of these networks (DCTN1 was identified as hub gene in the section of protein modification analysis).

### Relationship Between the ESAS and Immune Cell Infiltration

**Figure 5:**
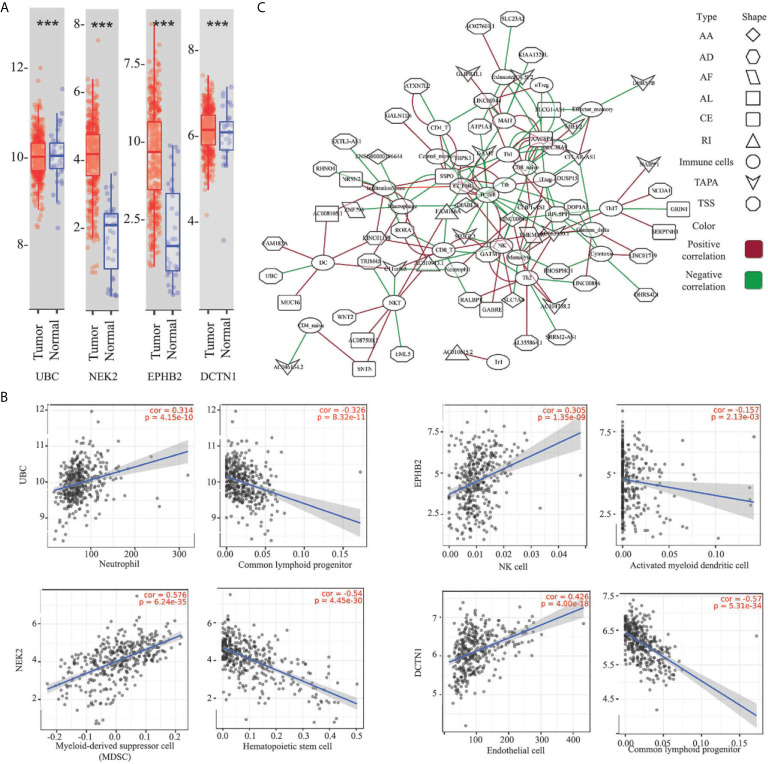
Relationship between the ESAS and immune cell infiltration. **(A)** Expressions of the UBC, NEK2, EPHB2, and DCTN1 in the TCGA STAD cohort (***p-value < 0.001); **(B)** Representative plots of the immune infiltration and the expressions of the UBC, NEK2, EPHB2, and DCTN1 in the TCGA STAD cohort; **(C)** Spearman’s rank correlation network of the ESAS and 24 types of immune cells (including the infiltration score). Red: positive correlation; Green: Negative correlation. ***p < 0.001.

To untangle the relationship between the hub genes and gastric tumorigenesis, we analyzed the hub genes in TCGA STAD dataset using the TIMER 2.0 database. We found that the UBC, NEK2, EPHB2, and DCTN1 genes were dysregulated in the TCGA STAD dataset (p-value <0.001, **Figure 5A**). The correlation between the expression of the hub genes and cell abundances in the STAD dataset was analyzed. We found that the expression of the UBC gene was positively correlated with the infiltration level of common lymphoid progenitor cells but was negatively correlated with the infiltration level of neutrophil cells. The expression of the NEK2 gene was positively correlated with the infiltration level of myeloid-derived suppressor cells (MDSC) but was negatively correlated with the infiltration level of hematopoietic stem cells. The expression of the EPHB2 gene was positively correlated with the infiltration level of NK cells but was negatively correlated with the infiltration level of activated myeloid dendritic cells. The expression of the DCTN1 gene was positively correlated with the infiltration level of endothelial cells but was negatively correlated with the infiltration level of common lymphoid progenitor cells (**Figure 5B** and **Table S7**). The above findings supported our conclusion that the hub genes played vital roles in the gastric tumorigenesis and indicated the potential relationships between the AS events and cell abundance.

Here, we postulated that the ESAS might be also involved in the immune cell infiltration. To test the hypothesis, the immune cell abundancy in the EOGC was calculated using the ImmuCellAI database. Then, Spearman’s rank correlation analyses were performed to indicate the relationship between the ESAS and immune cell infiltration in the EOGC. In total, 24 types of immune cells (including the infiltration score) were correlated with 77 ESAS events (Spearman’s rank correlation coefficients > 0.4 or < -0.4, BH adjusted p-value < 0.05, **Figure 5C** and **Table S8**).

### Network of the ESAS and Regulatory Factors

The AS events are regulated by various factors, including splicing factors (SFs) and APA core factors. However, it is unknown how the ESAS are regulated by these factors in the EOGC. To gain insights into this, we built the correlation network between the expression of 71 experimentally validated SFs, 22 APA core factors, and the PSI values of ESASs (27, 28).

**Figure 6:**
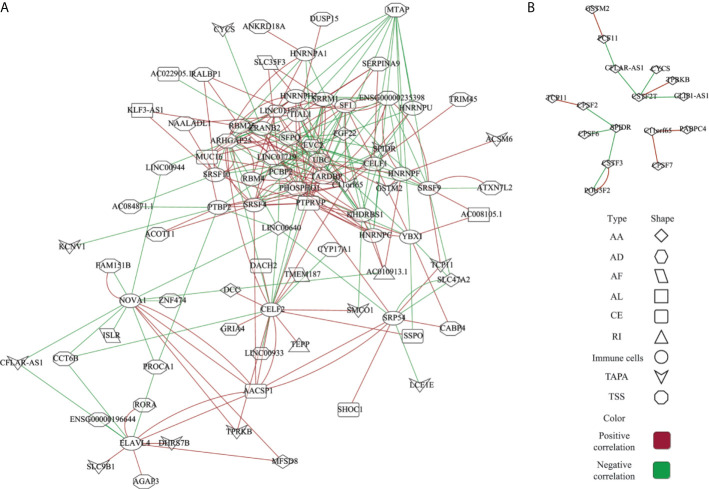
Network of the ESAS and regulatory factors. **(A)** Spearman’s rank correlation network of the ESAS and SFs; **(B)** Spearman’s rank correlation network of the TAPA and APA core factors. Red: positive correlation; Green: Negative correlation.

In the network of the ESAS and SFs, 70 ESASs were associated with 25 SFs (**Figure 6A** and **Table S9**). All SFs were significantly correlated with at least five AS events. Moreover, one AS event could be regulated by up to 28 different SFs. We also constructed a network of APA core factors and TAPAs, which included seven APA core factors and ten ESAS events, respectively (**Figure 6B** and **Table S9**).

### Clinically Relevant and Protein Modification Associated AS Events

**Figure 7:**
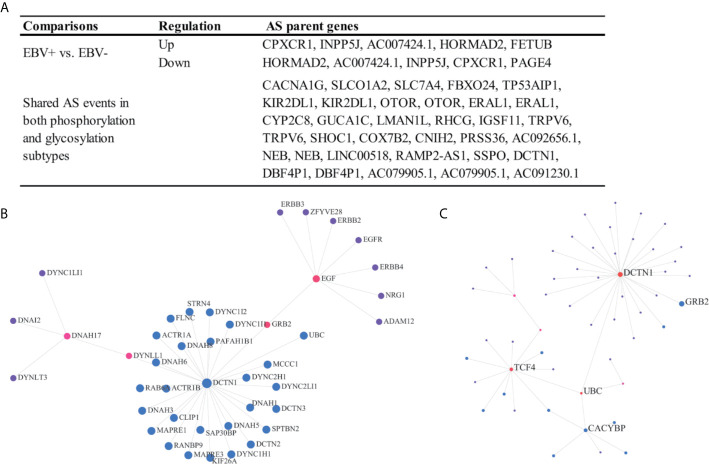
Clinically relevant and protein modification associated AS events. **(A)** Clinically relevant AS events; **(B, C)** Protein-protein interaction (first-order) networks of the glycosylation subtype and phosphorylation subtype related AS event parent genes, respectively.

There are few analyses to identify clinically relevant and protein modification associated AS events in the EOGC or GC. In this study, we identified 864 differentially expressed AS events from 577 genes between the EBV positive and negative tumor tissues (**Figure 7A**), 574 AS events from 533 genes associated with the MSI-High (MSI-H) and MSI-Low (MSI-L) tumor (**Table S10**).

The patients were subtyped according to the status of protein modifications, including phosphorylation and glycosylation, by a previous study (9). We found that 82 AS events from 60 genes were differentially expressed among the phosphorylation subtypes one to three, while the other 85 AS events from 65 genes were differentially expressed among the glycosylation subtypes one to three (**Figures 7B, C** and **Table S10**). In addition, a total of 27 AS events were dysregulated in both the phosphorylation and glycosylation subtypes (**Figure 7A**). The DCTN1 gene is a key hub gene in both phosphorylation and glycosylation networks.

## Discussion

Investigation of the AS events using the TCGA dataset has been a promising method and resulted in better understanding of the roles of the mRNA processing in the malignancy diseases (33–35). However, it remains challenging to reveal the roles of the AS events in certain subtypes of cancer (7). The unique subtype of cancer can be contributed by the AS events during the tumorigenesis. For example, the DOCK5 gene variant was identified as an oncogenic isoform in the HPV-negative head and neck squamous cell carcinoma (36). Herein, we conducted a systematic study to profile the AS events in the EOGC. In addition, we elucidated the roles of ESAS events with the splicing factors, APA core factors, immune cell infiltration, and protein modifications.

The distribution of the AS events in the gastric cancer was tissue-specific and related to the prognosis of patients (37). Furthermore, the expression patterns of the AS events in gastric cancer could be altered due to the Epstein-Barr virus infection (5). In this study, the RNA-Seq data with the protein modification status for subjects obtained from a published report were analyzed (9). In addition, we used Whippet to map the fastq format file to the reference genome, which generated two additional AS patterns (TSS and TAPA) than SpliceSeq (38) or MATS (39). These two approaches made the results more accurate and comprehensive than studies based on the TCGA SpliceSeq database (40).

According to our results, 267 ESAS, 6152 gene isoforms, and 4809 genes were aberrantly expressed in the EOGC patients. The parent genes of the ESAS were significantly enriched in the GO and KEGG. Further analyses indicated that the UBC, NEK2, EPHB2, and DCTN1 genes were the hub genes in PPI networks. It was reported that the UBC gene regulated the cell ubiquitin under normal and stressful conditions and the TSS events were identified within the promoter region of the UBC gene (41). The NEK2 protein was considered a splicing factor kinase through phosphorylation of the splicing factors, including the oncogenic SRSF1 protein (42). Alternative splicing and alternative polyadenylation encoded a variant of the EPHB2 gene which is a member of the EPH receptor protein-tyrosine kinase (43). The genetic structure variability of the DCTN1 gene was involved in the development of the limb-girdle muscular dystrophy (44) and neuron differentiation (45). These reports might shed light on the roles of the AS events and hub genes in the gastric cancer pathogenesis.

The distribution of tumor-infiltrating lymphocytes in the gastric cancer was correlated with the tumor histological type and clinical outcome (46). On the basis of the immune infiltration data, the immunoscore, a prognostic signature, was established for prognostic predictions of gastric cancer (47, 48). Our results showed that the hub genes and ESAS were correlated with distinct immune cell populations in the EOGC. For example, the TRIM45 gene encodes a protein to regulate the MAPK and NF-κB pathways which may inhibit the cancer cell proliferation (49). In our study, the TSS events of the TRIM 45 gene were positively correlated with macrophages but negatively correlated with NK T cells. Thus, we postulated that the alternative splicing of the genes might have potential roles in regulating the abundance of certain immune cells in the EOGC.

Two major types of regulatory factors, SFs and APA core factors, participated in the modulation of the AS events (15, 50). Hence, we built two networks (SFs vs. ESAS and APA core factors vs. TAPA) based on the Spearman’s rank correlation. We found that the dysregulation of SFs was highly associated with the ESAS expression and the APA core factors were linked to the TAPA events, which is consistent with the findings in previous reports (51, 52). Our results suggests that the SF and APA core factor contributes to the post-transcriptional mRNA processing of the EOGC.

Alternative splicing modulates the protein modification such as the protein phosphorylation (53) and protein glycosylation (54). The alternative splicing might modulate the protein modification through the isoform switch and change of the binding domain for N-linked glycosylation (55). One CD44 isoform has been shown to activate the RAS through the phosphorylation of the Erk (56). In addition, the small molecule, SM08502, reduced the phosphorylation of the splicing factors and generated the isoforms of the Wnt pathway genes which inhibited the gastrointestinal tumors (57). The reports about the alternative splicing and protein glycosylation are limited. Several studies focused on the CD44 variant 9 in the gastric cancer (58) and variant 6 in the colon cancer (59).

To our knowledge, the current study is the first to conduct a systematic analysis of the alternative splicing and protein modification based on the RNA sequence and mass spectrometry data in gastric cancer. The differential expressions of the AS events among the subtypes of the protein modification as well as EBV status and MSI status in the EOGC indicated that the AS events of the EOGC are subtype-specific. However, the shared AS events among different subtypes might demonstrate the close association of the subgroups defined by the protein modification or viral infection in the EOGC.

In conclusion, implementation of the rigorous criteria ensured the identification of the specific AS events related to the EOGC. In total, 267 ESAS events, 6152 gene isoforms, and 4809 genes were identified and might play vital roles in the EOGC tumorigenesis. The hub genes of the PPI networks and ESAS events might be valuable in deciphering the immune microenvironment in the early-onset gastric carcinogenesis. In addition, the SF and APA core factor correlation networks revealed the underlying pathways of the splicing modulation. Furthermore, a comprehensive analysis of the subtype-specific AS events suggested certain connection between the protein modification and viral infection in the EOGC. The findings in this study might be valuable in the clinical diagnosis and prediction of the early-onset gastric cancer.

## Data Availability Statement

Publicly available datasets were analyzed in this study. This data can be found here: https://www.ebi.ac.uk/ena/browser/view/PRJNA508414.

## Ethics Statement

Ethical review and approval was not required for the study on human participants in accordance with the local legislation and institutional requirements. Written informed consent for participation was not required for this study in accordance with the national legislation and the institutional requirements.

## Author Contributions

Designed the study and analyzed the data: JZ and LZ. Critical revision and structure suggestions: AG. All authors contributed to the article and approved the submitted version.

## Funding

The work was partially supported by the National Cancer Institute of the National Institutes of Health (R15CA213103) and Texas A&M University T3 grant (247099).

## Conflict of Interest

The authors declare that the research was conducted in the absence of any commercial or financial relationships that could be construed as a potential conflict of interest.
